# A database and tool, IM Browser, for exploring and integrating emerging gene and protein interaction data for *Drosophila*

**DOI:** 10.1186/1471-2105-7-195

**Published:** 2006-04-07

**Authors:** Svetlana Pacifico, Guozhen Liu, Stephen Guest, Jodi R Parrish, Farshad Fotouhi, Russell L Finley

**Affiliations:** 1 Center for Molecular Medicine and Genetics, Wayne State University School of Medicine, Detroit, MI 48201, USA; 2 Department of Computer Science, Wayne State University, Detroit, MI 48201, USA

## Abstract

**Background:**

Biological processes are mediated by networks of interacting genes and proteins. Efforts to map and understand these networks are resulting in the proliferation of interaction data derived from both experimental and computational techniques for a number of organisms. The volume of this data combined with the variety of specific forms it can take has created a need for comprehensive databases that include all of the available data sets, and for exploration tools to facilitate data integration and analysis. One powerful paradigm for the navigation and analysis of interaction data is an interaction graph or map that represents proteins or genes as nodes linked by interactions. Several programs have been developed for graphical representation and analysis of interaction data, yet there remains a need for alternative programs that can provide casual users with rapid easy access to many existing and emerging data sets.

**Description:**

Here we describe a comprehensive database of *Drosophila *gene and protein interactions collected from a variety of sources, including low and high throughput screens, genetic interactions, and computational predictions. We also present a program for exploring multiple interaction data sets and for combining data from different sources. The program, referred to as the Interaction Map (IM) Browser, is a web-based application for searching and visualizing interaction data stored in a relational database system. Use of the application requires no downloads and minimal user configuration or training, thereby enabling rapid initial access to interaction data. IM Browser was designed to readily accommodate and integrate new types of interaction data as it becomes available. Moreover, all information associated with interaction measurements or predictions and the genes or proteins involved are accessible to the user. This allows combined searches and analyses based on either common or technique-specific attributes. The data can be visualized as an editable graph and all or part of the data can be downloaded for further analysis with other tools for specific applications.  The database is available at

**Conclusion:**

The *Drosophila *Interactions Database described here places a variety of disparate data into one easily accessible location. The database has a simple structure that maintains all relevant information about how each interaction was determined. The IM Browser provides easy, complete access to this database and could readily be used to publish other sets of interaction data. By providing access to all of the available information from a variety of data types, the program will also facilitate advanced computational analyses.

## Background

Genome sequencing and analysis projects have revealed thousands of genes with unknown or poorly characterized functions. A valuable approach to understanding the roles that novel genes play in normal biology or disease is to identify the intermolecular interactions in which they and their encoded proteins are involved. Groups of genes or proteins that are connected by intermolecular interactions often function together to mediate specific biological processes, such as DNA synthesis or the cellular response to environmental cues. A group of genes, for example, may encode proteins that interact with each other to form a regulatory pathway or to constitute a molecular machine that performs an enzymatic activity. Establishing the links between sets of genes or their encoded proteins can provide initial clues about the functions of individual poorly characterized genes, for example, by associating them with groups of genes with known functions. Linking genes into functional groups can also reveal insights into how they work together to mediate specific biological processes and can lead to a deeper understanding of those processes.

Several technologies have been developed to discover interactions between genes or their protein products, and some of these technologies have been scaled up with the ultimate goal of mapping all of the interactions encoded by a genome [1-3]. One of these technologies is the yeast two-hybrid system, which detects physical binary interactions between pairs of proteins [4]. High throughout two-hybrid screens have detected thousands of protein-protein interactions for various organisms, accounting for most of the interactions currently available in public databases [5-10]. A second technique that has been used in large-scale screens is co-affinity purification (co-AP) of proteins that are stably associated with individual query proteins, followed by identification of the co-purified proteins by mass spectrometry (MS) [11-13]. The result of such a co-AP/MS experiment is the identification of a group of proteins that may exist together in a complex in the cell. These studies have produced large data sets that have proven useful in expanding our understanding of previously identified protein interaction networks as well as in identifying biological networks that were previously unknown.

A drawback of the large-scale protein interaction studies however, is that they contain a relatively high number of false positives and false negatives. One successful strategy for overcoming this drawback is to simultaneously analyze multiple interaction data sets [8,14-16]. Combining data sets for a given organism can provide a more comprehensive view of the possible interactions for any set of proteins. It also reveals interactions that were observed in more than one study; these interactions, whether they are identified by similar or disparate methods, have been shown to be more likely to be biologically relevant, true positives. In addition to protein interaction data, other large-scale data sets that relate genes or proteins to one another can also be integrated to further enhance the power of this approach. For example, large-scale data sets are available that link genes to one another based on similar phenotypes following RNAi knock down or based on genetic interactions, which are altered phenotypes that result when alleles of two different genes are brought together into one organism [17-19]. Integrating these additional data sets with the protein interaction data can help reveal groups of proteins that function together (e.g., refs[18,20]). Finally, the development of increasingly accurate computational approaches has begun to produce sets of predicted interactions useful for generating testable hypotheses about the functions of proteins and pathways [21-27]. Several central repositories have become available for housing experimentally and computationally determined interactions [28-35]. Unfortunately, however, interaction data sets have begun to emerge so quickly and from such different sources that it has become difficult to find all of the relevant data in one location. Moreover, each set of interaction data has a unique set of attributes, some of which may be important for proper interpretations and analyses, but which are often discarded when the data is placed into a generic interaction database.

We set out to assemble all available gene and protein interaction data for *Drosophila *into a single, web-accessible database that includes all of the relevant attributes of each data set, and that can be readily updated with new data sets as they become available. We also developed a program, IM Browser, for browsing this or similar databases. The program was designed to be web-based, easy to use, require no special programs, be accessible from a variety of platforms, and allow the data to be searched, viewed, analyzed, saved, and downloaded in convenient forms. The program minimizes restrictions on data structure so that new types of interaction data can be readily accessed with minimal prior formatting. Powerful and rapid search and filter functions can be performed based on any attribute that is associated with a node (gene or protein) or an edge (interaction) in the data sets. Finally, the IM Browser, when combined with the *Drosophila *Interactions Database presented here, allows users to rapidly and easily integrate multiple data sets.

## Construction and content

### A database of *Drosophila *gene and protein interactions from multiple sources

We adopted a simple database structure with tables for two types of data: interaction data and gene/protein data. Tables for interaction data contain two fields that uniquely identify the two interacting genes or proteins. Interaction tables may also contain any number of additional fields, considered as interaction attributes. These attributes may include the type of experiment, the reference, and the various measured parameters for each interaction. Data sets with different attributes are placed into separate interaction tables. Each gene/protein table contains a field that uniquely identifies a node (gene or protein) and any number of additional searchable fields for gene/protein attributes. The gene/protein tables may also contain a field for a node label to be displayed on the graph, and a URL linking to an external database of gene/protein information. In the *Drosophila *Interactions Database we used the Flybase Gene Number (FBgn) from the Flybase database [36,37] to uniquely identify each gene/protein, and pairs of FBgns to uniquely identify each interaction. We implemented this database in Oracle 9i. The database schema can be found in the supplemental figure [see Additional file 1].

The *Drosophila *Interactions Database described here currently contains six interaction tables. Two of the tables contain *Drosophila *protein-protein interactions that were predicted based on interactions detected between orthologs in either *C. elegans *or *S. cerevisiae*. These interactions have been referred to as 'interologs' [38,39]. The *C. elegans *interactions were from a large two-hybrid screen [7], while the *S. cerevisiae *interactions were from a consolidated database [40] predominated by data from large-scale two-hybrid [6,9] and co-AP/MS [11,12] experiments. Another table contains genetic interactions downloaded from Flybase. Genetic interactions are detected between alleles of two genes and often suggest that the genes function in the same or parallel pathways [41]. Three tables contain *Drosophila *protein-protein interactions derived from different high throughput yeast two-hybrid screens. One contains published data from a group led by researchers at Curagen [5], the second contains published data from a group at Hybrigenics [10], and the third contains interactions from our published [8] and ongoing work. While all of these tables contain yeast two-hybrid data, they differ significantly in the type of information that was collected about each interaction. In the data from Giot et al. [5], for example, each interaction was assigned a confidence score based on a system that was unique to that data set; thus, the other two data sets have no comparable score. In the data from our two-hybrid screens, on the other hand, two-hybrid reporter activity scores were recorded for each interaction, and no similar scores were obtained by the other two groups. These differences illustrate the value of a flexible database structure and interface. Rather than combining the data into one generic table, which might require discarding some data or lumping the incompatible attributes into one "other" field, we chose to put each of the different data types into its own table with fields for every attribute of that data type. The IM Browser described below is able to dynamically access all of the different attributes in existing tables or from newly added tables. The database also contains a number of gene/protein or node information tables. These include tables with gene annotation data obtained from Flybase, including the Gene Ontology [42] classifications, Molecular Function, Biological Process, and Cellular Component, the Flybase URL, gene name, synonyms, protein domains, protein sequence, and cytogenetic map location.

## Utility

### Implementation of an interaction data browsing tool

We developed the IM Browser as a web-based application to navigate gene and protein interaction data stored in multiple tables of a remote relational database system. The program accesses a database defined by the user, lets the user select tables of interest, reads from the database schema of the selected tables, and generates a graphical interface for building queries; results of the queries are integrated in a single graph, where nodes represent the genes or proteins and edges connecting nodes represent the interactions. We developed the IM Browser with a three-tier system architecture consisting of Oracle database technologies, a Java servlet using the yFiles graph library (yWorks, Tübingen, Germany), and a Java applet running on the user's computer. While our tool was designed and tested with Oracle 9i, it could be customized to work with other Relational Database Management Systems (RDMS) that understand SQL commands.

The IM Browser program is designed to access a database in three different ways. First, the program has the ability to start with a default database connection. When IM Browser starts, it looks for a description file with a configuration of the default database. If the file is found, IM Browser connects to the specified database and displays names of tables ready to be queried. We have set up an instance of the program [43] with a default connection to the database containing interactions and gene/protein information for *Drosophila *described above. Second, in the absence of a default database connection file, or to connect to a different database, the program allows users to specify the database and define which data tables will be presented and searchable, which attributes in the tables will be used to specify the nodes (genes or proteins), and which attributes will be used to label nodes on the graph. To make a new connection, the user specifies a database (e.g., URL), name, login ID, and password. After the servlet successfully connects to the database server, IM Browser displays the names of available tables and lets the user select the tables of interest. The user must specify whether the table contains either interaction data or gene/protein/node attribute data. At any time during the session the user can add more tables from the same database. Third, users can load a database connection configuration file that was saved from a previous successful connection. The database connection configuration file can be saved during any session to the users local computer as an XML file, and the file can be shared with other users.

### Generating interaction maps from multiple data sets

**Figure 1:**
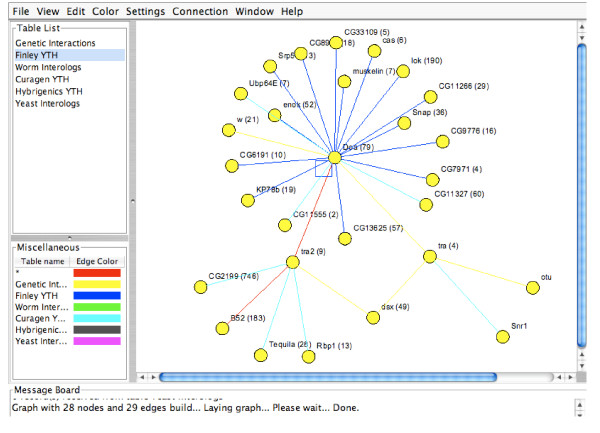
**A typical IM Browser window**. The panel at the left lists the interaction tables available in the database. New tables added to the database are dynamically added to the list. The main window (right) shows an interaction map with nodes (yellow circles) representing genes or proteins and edges connecting them representing interactions. The edges are colored based on the tables from which the data came, according to the key in the lower left panel; in this case, red edges indicate that the interaction is found in at least two interaction tables. The coloring scheme can be defined by the user as described in the text. Nodes and edges in the graph are both 'clickable' to obtain more information about the gene/proteins or interaction attributes from the data tables, respectively, and to link to outside databases. The graph can be manipulated manually or redrawn based on preset or user-defined parameters.

**Figure 2:**
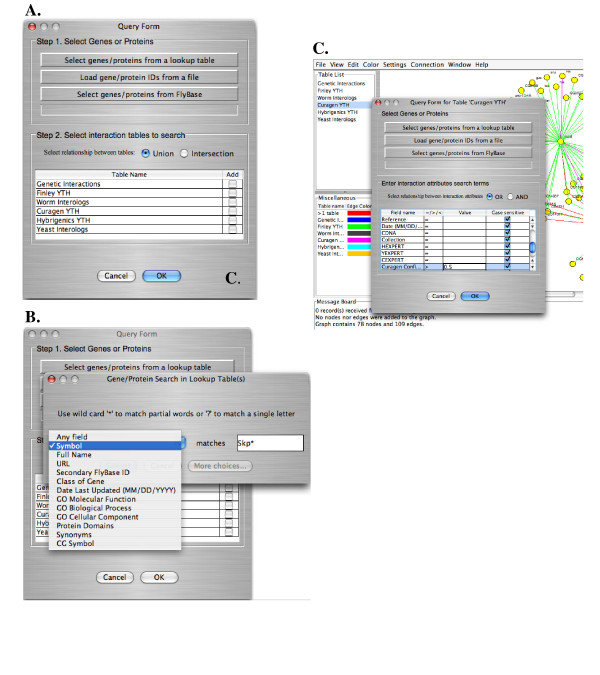
**Examples of ways to query the database with IM Browser**. (A) The query form for adding new interactions to an existing graph, drawing a new graph, finding nodes to color, or applying filters to an existing graph. In Step 1, genes or proteins are selected to be used in a search of the interaction database. To select genes or proteins, gene identifiers (IDs) or wild-cards (*) can be entered or looked up from either Flybase or a lookup table based on gene/protein attributes. A text file of gene IDs can also be uploaded. In Step 2, the interaction data tables to search are selected by checking boxes in the lower half of the window, and the relationship (Union or Intersection) between the interaction tables is specified. (B) Searches can be performed on any or all attributes in the gene/protein information or "look up" table(s). All of the attributes from these tables are listed and searchable; attributes in newly added tables are dynamically added to the list. (C) Individual interaction tables can also be searched to find new interactions or to apply filters to an existing interaction map. All of the attributes in each interaction table are searchable with operators ('=', '<', '>'), and the relationship ('AND', 'OR') between attributes can be specified by the user. In the example, a search of the 'Curagen YTH' table for interactions with a confidence score >0.5 is about to be initiated.

Figure 1 shows a typical screen shot of the IM Browser window. The window is divided into four panels. The top left panel shows the list of interaction tables from the database that are available for queries, the bottom left panel provides a key to the current coloring scheme, the right panel shows the interaction map, and the bottom panel is a message board showing the current number of nodes and edges in the map and the results of recent manipulations. A new interaction map can be generated by selecting 'New Graph' from the 'File' menu, or new interactions can be added to the current map by selecting 'Add Gene/Protein' from the 'Edit' menu. In each case, a window opens as in Figure 2A to allow users to search the database.

A key feature of the IM Browser is that it provides a user-friendly interface to take advantage of the powerful search engines in the RDMS. Users have several options for generating maps relevant to their interests. Users can search the database by gene/protein attributes, by interaction attributes, by gene/protein IDs or by any combination of these. Several of the search options are available from the Query Form (Fig. 2A), which is available when creating a new graph or adding interactions to an existing graph. First, users can search on any one of the gene or protein attributes found in gene/protein information lookup tables in the database. Every field of each gene/protein attribute table is accessible for searching; if new tables with gene or protein information are added to the database, their fields are dynamically added to the list of available attributes that can be searched (Fig. 2B). Second, users can combine gene or protein attributes by joining search terms with "AND" or "OR" statements. The program provides easy pull-down menus and check boxes to generate simple or complex searches. Third, users can directly enter one or more gene identifiers for the gene(s) or protein(s) of interest, or upload a list of gene identifiers as a text file. This approach is particularly useful, for example, to search the database with a list of genes obtained as output from another analysis program or from database searches. Fourth, in the instance of IM Browser that we set up for the *Drosophila *Interactions Database, users can connect directly to Flybase and search the extensive information available there to find genes or proteins with the desired properties to add to the interaction maps (Fig. 2B). Finally, users can enter a wild card "*" as the gene identifier and obtain all of the interactions in a particular table or combination of tables.

Once the genes of interest are entered or uploaded, the user selects the interaction tables to be queried. The user can select a single interaction table or join two or more tables (Fig. 2A). With the join relation "Union" the interactions that are found in at least one of the selected tables will be extracted and presented. With the join relation "Intersection" only interactions that are common to the selected tables will be extracted and presented. These options provide the user with the ability to take a comprehensive look at all available data pertaining to their gene(s) of interest, or to focus on interactions detected in multiple different selected datasets. In addition to searches based on gene or protein attributes, users can conduct searches based on any of the attributes found in the interaction tables. This is achieved by selecting one of the tables from the Table list in the upper left panel, which produces a list of all searchable attributes for that dataset (Fig. 2C). Search values can be entered into one or more fields and the fields can be joined by "AND" or "OR" relationships. This feature provides searchable access to all of the information in every dataset, even though different datasets often have disparate attributes.

The data returned from a search is displayed as an interaction map in a default format that can be manipulated in a number of ways, as described further below. The data can also be saved in three different formats. An image of the graph can be saved to the user's computer in GIF format at a resolution chosen by the user. The raw data can also be saved in a tabular format as a list of interactions and their attributes. When "Save Summary Table" is selected, the user is presented with a list of attributes from the source tables for the data being browsed. The user can select any subset or all of the attributes to be saved to the table on the local computer. This facilitates further downstream analyses using other specialized methods or programs. Finally, the results of the user's search can be saved to the local computer in a "PIM" format that can be reloaded later. In the PIM format, IM Browser saves the database connection configuration and all of the user's actions that resulted in the currently displayed data. These actions may include successive searches to add new genes or interactions to the map, or the deletion of nodes either individually or in sets, for example, as a result of applying a filter, as described further below. While reading a saved PIM file, IM Browser connects to the same database and reruns the user's actions. If the data in the database did not change since the PIM file was saved, the same graph will be created again and its analysis can be continued. If the data in the database was modified, the graph will reflect the changes. Thus, the saved session allows further analysis and updating of a particular network at any time from anywhere on the Internet. This format also promotes the sharing of data mining protocols, which can be executed periodically on data that may be updated frequently.

### Mining interaction maps for biological insights

An interaction map can serve as a convenient starting point for generating hypotheses about the functions of genes and pathways. To aid in this process the IM Browser has several features for further exploring and manipulating interaction maps. A few of these features will be discussed here, while a more complete list and explanation of all features can be found in the help file [43]. Once an initial map has been created, new interactions can be added or deleted based on properties of the interactions or of the nodes. Individual nodes can be manually deleted or they can be expanded to show all of their interactions from the database. Nodes can also be added by searching for particular gene/protein attributes as described in the previous section. Nodes can be deleted from the map by applying a filter to the data. The filter function allows users to find and delete nodes based on their properties. Genes or proteins having a particular functional annotation or with a particular type of domain, for example, can be found and deleted. Nodes can also be deleted based on their number of interactions, allowing users to filter out promiscuous proteins at a user-defined threshold. This functionality can also be used to focus on more highly connected regions of a particular network by successively filtering out singly connected nodes. For each filter, the program finds the genes/proteins based on the user's search criteria, highlights them on the map, and allows the user to either cancel or proceed with the deletion. This enables users to explore different potential subnetworks of a map and experiment with the removal of particular classes of genes or proteins.

Several features of the IM Browser allow users to find and focus attention on regions of an interaction map that may be of particular biological interest. First, the general layout of the map can be adjusted either manually or via preset layout modes to highlight different features of the map. For example, the hierarchical and circular layouts can reveal topographical features of the map that are difficult to discern from a random layout of the nodes, including hub proteins and clusters. These and other topoplogical properties have been shown to correlate with biological properties of the genes or proteins and their interactions [44]. Second, nodes and edges can be manually colored or sized to highlight their potential importance and to keep track of them as interactions are added or deleted or as the map layout is manipulated. Third, edges can be colored based on the data sets from which they are derived. This feature is particularly useful as it makes integration of multiple interaction data sets evident from the map. For example, interactions can be colored based on the number of different interaction tables that contain them. Many studies indicate that interactions found in multiple independent data sets are less likely to be false positives than interactions found in a single data set, which may be artifacts of a particular screen or technology; therefore, interactions found in multiple data sets are more likely to be biologically relevant [8,15,16]. Fourth, the nodes that are found based on a search of gene/protein attributes can be colored rather than deleted. This is particularly useful for marking genes or proteins based on their known functional annotations. A key finding from analyses of numerous protein interaction maps is that the function of a protein can be inferred with some accuracy based on the functions of the proteins with which it interacts. Moreover, the accuracy of the prediction increases as the number of connected proteins with the same function increases. The underlying finding that supports this "guilt-by-association" method is that clusters of interconnected proteins often participate in the same biological process [45-48]. While sophisticated approaches have been developed for finding these clusters, they can also become evident to the casual browser of an interaction map, particularly by coloring proteins with shared functions.

Edges can also be filtered from an interaction map based on properties of the interactions. This is accomplished by entering search values for any one or combination of attributes found in the interaction data tables (Fig. 2C). The results of the search are highlighted in the interaction map and the user is given the opportunity to abort the filter or to proceed with deletion of the highlighted edges. One example of how such filters can be useful is for interaction data that has confidence scores associated with each interaction. Various confidence scoring systems have been devised to assign a score to each interaction in a dataset indicating the likelihood that the interaction is biologically relevant [5,10,49,50]. Unfortunately, no universal system has been developed, and therefore, the best that a confidence scoring system can do is to provide an internally consistent measure of the relative quality of interactions within a particular data set. Thus, when analyzing multiple data sets via an interaction map, it is important to be able to access the attributes of each data set independently and to be able to apply filters for confidence levels based on different scales. Not only do different data sets have different attributes, they may also have different biases. The ability to apply filters based on combinations of attributes across multiple data sets provides biologists with unlimited flexibility to find and focus attention on regions of an interaction map that are the most important for any particular study.

**Figure 3:**
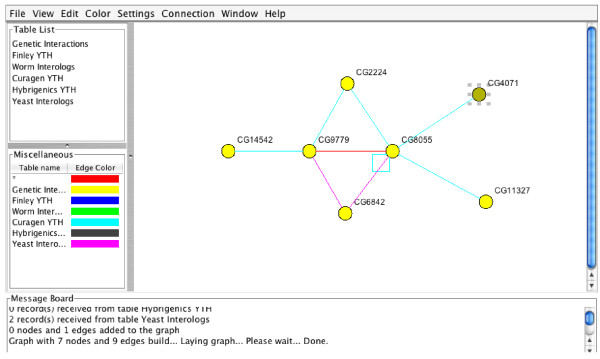
**A protein cluster evident from combined data sets**. The cluster involving *Drosophila *proteins (CG2224, CG9779, CG8055, CG6842) includes interactions from the 'Curagen YTH' table and the 'Yeast Interologs' table. The interaction between CG9779 and CG8055 was detected in both tables, and thus might be considered a higher confidence interaction. The graph also shows that CG8055 interacts with itself (blue box).

While interactions detected in two or more high throughput screens can often be assigned higher biological significance, the generally low coverage of these screens has minimized the opportunities for such cross validation. The data from three large-scale two-hybrid screens in *Drosophila *illustrates this problem [5,8,10]. Combined, the three screens resulted in 24,121 interactions, and yet only 57 were detected in any two screens, and only one was detected in all three. Thus, until screens reach saturation or screening approaches and technologies improve, biologists will need to combine data sets to get the most complete picture of an interactome possible. The *Drosophila *Interactions Database along with the IM Browser combines data sets and allows users to view the high confidence interactions in the context of all available interactions. Figure 3 shows an example where the confidence of interactions is not only increased by direct overlap of two data sets, but also by topographical features that are only evident when the two data sets are combined. In this case, the topological feature is a cluster of interacting proteins.

**Figure 4:**
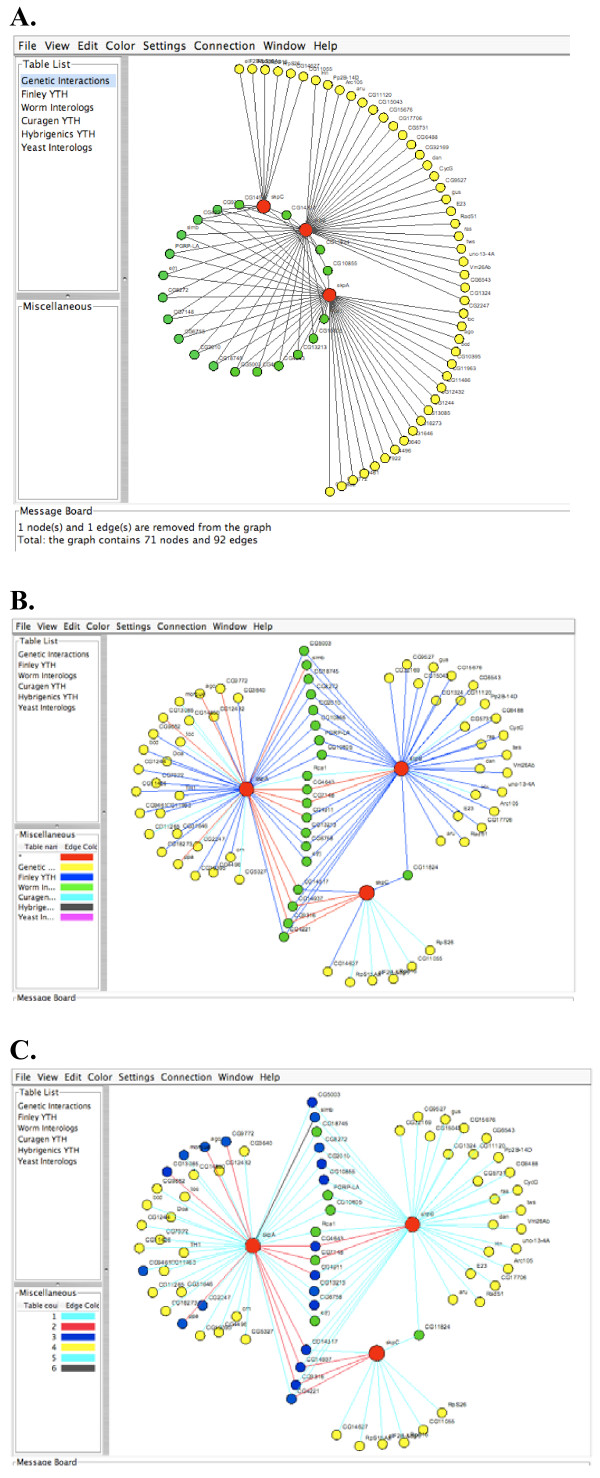
**Mining interaction data for biological insights**. (A) Circular layout of a map generated by searching the *Drosophila *Interactions Database for interactions involving 'skp' proteins (colored red). Proteins that interact with two or more Skp proteins are on the circle and were colored green. (B) The map in (A) was redrawn to an 'organic' layout and the edges were colored based on the tables from which the interactions were found. The red edges show interactions found in two or more data sets. (C) The database was searched for proteins that have F-box domains and these were colored blue. Note that a preponderance of the blue nodes can be found in the central group of nodes that interact with two or more Skp proteins.

In Figure 4 we show an example of how the IM Browser might be used to find a biologically important subnetwork from which testable hypotheses might be generated regarding protein and pathway function. First we simultaneously searched three different large scale yeast two-hybrid data sets to find interactions involving members of the Skp family of proteins, which are involved in targeting many different proteins for ubiquitin-mediated proteolysis [51]. The three Skp proteins in the map were then identified by searching for nodes in which "Skp" was found in the 'gene symbol' or 'synonym' attributes of the gene/protein attribute table, and these nodes were enlarged and colored red. The circular layout was then used to easily visualize nodes that were connected to multiple Skp proteins, and these nodes were colored green (Fig. 4A). Next, we changed to an organic layout and colored the edges based on the interaction data sets in which they were detected (Fig. 4B). This illustrates that a comprehensive view of the potential Skp pathway members requires combining data from the different two-hybrid screens, since each single data set misses several potentially important interactions. Next, we searched the gene/protein attribute table and Flybase directly to find proteins with F-box domains, which are known to interact with Skp proteins [52], and colored the corresponding nodes blue. As previously noted [8], most of the F-box proteins in the map connect to multiple Skp proteins, suggesting that this topological position is enriched for proteins that play a role in the Skp pathway. Finally, we used the color edge feature to color the edges based on the number of data sets in which they are found. This revealed that 16 interactions were detected by at least two independent screens (Fig. 4C). Interestingly, all of these interactions involve proteins with F-box domains, and thus are expected interactions, consistent with the hypothesis that interactions detected in multiple high throughput screens are more likely to be true positives than those detected in only one screen. The map also shows that one interaction (SkpA-Slmb) was detected in all three two-hybrid screens. In the "Curagen" data set, this interaction had only received a confidence score of 0.42, below the 0.5 considered to be the dividing line between high confidence and low confidence [5]. Such interactions would be missed by initially focusing on only high confidence interactions. In the context of the interaction map from multiple data sets, however, the interest in this interaction would be boosted by its topological position (interacting with two Skps), its domain structure (F-box), and the fact that it was detected in several independent screens.

## Discussion

Efforts to understand how genes and their encoded proteins work together to mediate biological processes have become a central focus and challenge of current biological research. Maps that depict the physical and functional interactions among genes and their protein products are useful starting points for developing a systems level understanding of biological processes. The usefulness of these maps, however, depends on the comprehensiveness and quality of the experimental and computational data underlying them. While many technologies have been developed to attempt to collect this data on a genome-wide or proteome-wide scale, all suffer from relatively poor coverage of the possible interactions and confounding rates of false positives. Thus, it has become clear that maximally useful interaction maps must be derived from the combination and integration of all available data sets. To aid in this endeavor, we set out to create a comprehensive publicly accessible database that assembles all of the interaction data available for *Drosophila *into one location. We also developed a web-accessible interface, IM Browser, to facilitate mining this and related databases.

Several public databases have been developed in recent years to collect and present gene or protein interaction data [28-35]. While the data in these is massive in terms of the number of interactions and the number of different organisms represented, it is also only partially redundant, requiring biologists to consult each of them to ensure that all relevant data has been obtained for the genes or proteins under study. This requires users to negotiate several different interfaces, and often, to manually pick out the non-overlapping data and assemble it into a single interaction map using, for example, a mapping program like Cytoscape [53]. We wished to simplify this process for researchers interested in the model organism *Drosophila*. We focused our attention on constructing a database that would present interaction data for *Drosophila *genes and proteins. We have included data derived from both experimental and computational approaches, including our own data and that taken from other published work and central databases. We have also begun to integrate interaction data from other organisms by presenting interolog tables, which represent how the interactions from other organisms may correspond to interactions among *Drosophila *proteins. Finally, we set out to preserve all of the features of the interaction data and make them accessible for analyses. Many databases currently either do not accommodate all interaction attributes, or for convenience do not store or make them accessible. Making this information available will foster development of new computational approaches to extract biological meaning from the data.

A convenient way of representing interaction data is in the form of a graph or a map, in which nodes represent genes or proteins while the edges connecting nodes represent the interactions. Unlike lists of interactions, maps show individual genes or proteins in the context of their surrounding interaction network. This enables biologists to readily navigate from one region of a network to another. Maps also provide graphical representations of topological features that may have biological relevance. Thus, interaction maps provide not only a convenient interface for browsing interaction data, but also a formal framework for understanding biological processes. Several programs have been developed for visualizing and browsing specific interaction databases [10,53-55]. While a few of these afford powerful tools for analysis there is still a need for highly accessible, user-friendly programs that enable complete access to all relevant data sets and to new data as it becomes available. IM Browser is an alternative interaction data visualization and exploration program particularly well-suited for databases with multiple interaction data sets. Although we developed IM Browser to access our combined *Drosophila *Interactions Database, the program could readily be used to access or publish other organism-specific databases as well.

Combining the interaction data for one organism into one database provides not only the most comprehensive view of an interactome possible, but also facilitates analyses to distinguish false positives from biologically relevant interactions. For example, several studies have shown that interactions detected in multiple screens or by different technologies are more likely to be biologically relevant than those detected in only one experiment, and this is particularly true for high throughput data [8,15,16]. The IM Browser facilitates easy visualization of these interactions, either alone or in the context of all interactions. A second approach to focusing on the interactions with the highest biological significance is to utilize the confidence scores given to individual data sets. The IM Browser and *Drosophila *Interactions Database facilitate this by including all of the confidence scores available and by allowing searches that combine the scores from different data sets with simple logical operators. In addition, providing access to all of the individual experimental and computational evidence for interactions is likely to facilitate the development of better confidence scoring systems. In the confidence scoring approach used by Giot et al. [5], attributes of the interaction data were used to train a statistical model to determine the likelihood that a particular combination of attribute values correlate with known high or low confidence interactions. This enabled each interaction to be assigned a probability score based on its attributes. A similar approach could be taken with each data set or with combinations of data sets. Moreover, by combining the probabilities for each data set, a combined confidence score could be derived, as has been suggested by Fraser and Marcotte [56]. Thus, the IM Browser provides the tools needed to integrate multiple data sets into a single map and to guide biologists toward the most promising data for further study. Thus, the program and database described here offer an alternative starting point for analyzing protein networks and discovering protein and pathway function.

## Availability and requirements

Access to the *Drosophila *Interactions Database and the IM Browser is freely available through the website . The database is accessible by using the IM Browser, which requires an internet connection and a web browser with a Java plug in. The program has been tested extensively and works well using Internet Explorer version 6 on a Windows XP system, and Safari on an OS X system, with Java plug-in 4.0 or higher installed. The IM Browser can also be used to download all database tables in tab-delimited text format. The code for the IM Browser is also available upon request.

## Authors' contributions

SP did all Java programming and interface development. GL compiled the interactions databases. SG and JRP helped with interface development and beta testing. FF advised on java programming and database development. RLF guided database and interface development. All authors contributed to writing the manuscript.

## Supplementary Material

Additional File 1 ***Drosophila Interactions *Database table schema**. The database consists of two table types, the gene tables on the left and the interaction tables on the right. The gene tables currently include the Fly Gene Attributes table and the Fly Gene Expression table. Gene records in these tables are uniquely identified by their Gene IDs (Flybase_ID, also known as Flybase gene number or FBgn). As described in the text, the interaction tables currently include three tables of predicted binary interactions (Predicted Worm Interologs, Predicted Yeast Interologs, and Genetic Interactions), and three tables for interactions experimentally determined by yeast two-hybrid (YTH) assays (Finley Lab YTH, Curagen YTH, and Hybrigenics YTH). Interaction records are uniquely identified by pairs of Gene IDs. In the case of the predicted interactions, the interaction has no direction or orientation, and there is no significance to whether a gene is listed as "Gene 1" or Gene 2"; each pair of genes is uniquely listed in only one, arbitrary orientation. In the case of the YTH data, on the other hand, each measurement is made with a gene as either the "BD" or the "AD", and thus each interaction has a direction or orientation. Each pair of Gene IDs can be detected as interacting in the BD-AD orientation, the AD-BD orientation, or both. All of the interaction tables share certain common attributes, such as Data_version, Reference, and fields for the number of interactions for each gene. Each table also has table-specific attributes. Definitions of the attributes are available at . All attributes are searchable in IM Browser. Click here for file
