# Supplementary material for: A database and tool, IM Browser, for exploring and integrating emerging gene and protein interaction data for Drosophila
Source: BMC Bioinformatics. 2006 Apr 7;7:195. doi: 10.1186/1471-2105-7-195 (PMC1458360; doi:10.1186/1471-2105-7-195)
Supplement: Additional File 1 — Drosophila Interactions Database table schema. The database consists of two table types, the gene tables on the left and the interaction tables on the right. The gene tables currently include the Fly Gene Attributes table and the Fly Gene Expression table. Gene records in these tables are uniquely identified by their Gene IDs (Flybase_ID, also known as Flybase gene number or FBgn). As described in the text, the interaction tables currently include three tables of predicted binary interactions (Predicted Worm Interologs, Predicted Yeast Interologs, and Genetic Interactions), and three tables for interactions experimentally determined by yeast two-hybrid (YTH) assays (Finley Lab YTH, Curagen YTH, and Hybrigenics YTH). Interaction records are uniquely identified by pairs of Gene IDs. In the case of the predicted interactions, the interaction has no direction or orientation, and there is no significance to whether a gene is listed as "Gene 1" or Gene 2"; each pair of genes is uniquely listed in only one, arbitrary orientation. In the case of the YTH data, on the other hand, each measurement is made with a gene as either the "BD" or the "AD", and thus each interaction has a direction or orientation. Each pair of Gene IDs can be detected as interacting in the BD-AD orientation, the AD-BD orientation, or both. All of the interaction tables share certain common attributes, such as Data_version, Reference, and fields for the number of interactions for each gene. Each table also has table-specific attributes. Definitions of the attributes are available at . All attributes are searchable in IM Browser. [file 1471-2105-7-195-S1.pdf]

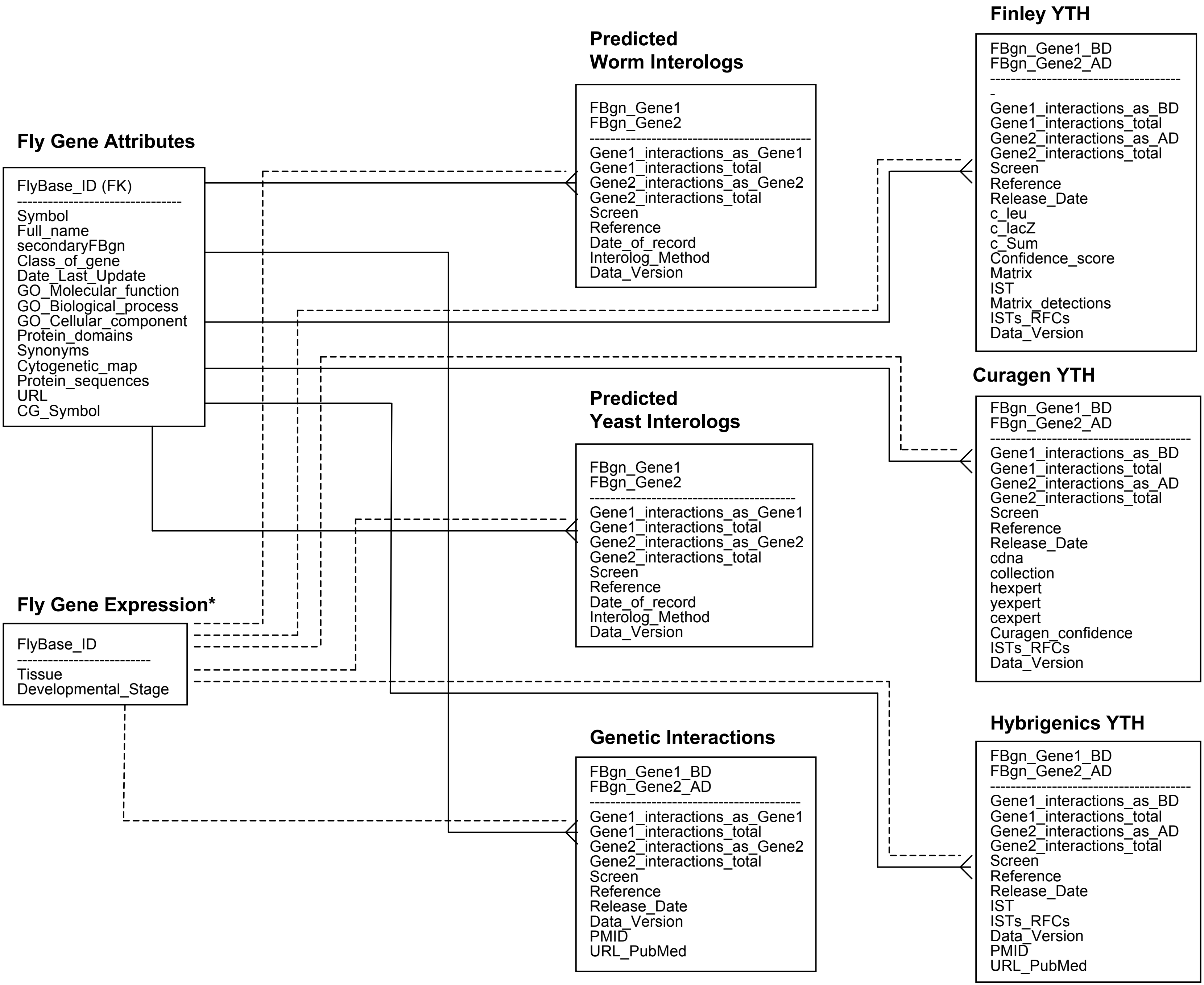
Fly Gene Attributes
FlyBase_ID (FK)

Symbol
Full_name
secondaryFBgn
Class_of_gene
Date_Last_Update
GO_Molecular_function
GO_Biological_process
GO_Cellular_component
Protein_domains
Synonyms
Cytogenetic_map
Protein_sequences
URL
CG_Symbol
Fly Gene Expression*
FlyBase_ID

Tissue
Developmental_Stage
Predicted Worm Interologs
FBgn_Gene1
FBgn_Gene2

Gene1_interactions_as_Gene1
Gene1_interactions_total
Gene2_interactions_as_Gene2
Gene2_interactions_total
Screen
Reference
Date_of_record
Interolog_Method
Data_Version
Predicted Yeast Interologs
FBgn_Gene1
FBgn_Gene2

Gene1_interactions_as_Gene1
Gene1_interactions_total
Gene2_interactions_as_Gene2
Gene2_interactions_total
Screen
Reference
Date_of_record
Interolog_Method
Data_Version
Genetic Interactions
FBgn_Gene1_BD
FBgn_Gene2_AD

Gene1_interactions_as_Gene1
Gene1_interactions_total
Gene2_interactions_as_Gene2
Gene2_interactions_total
Screen
Reference
Release_Date
Data_Version
PMID
URL_PubMed
Finley YTH
FBgn_Gene1_BD
FBgn_Gene2_AD

-
Gene1_interactions_as_BD
Gene1_interactions_total
Gene2_interactions_as_AD
Gene2_interactions_total
Screen
Reference
Release_Date
c_leu
c_lacZ
c_Sum
Confidence_score
Matrix
IST
Matrix_detections
ISTs_RFCs
Data_Version
Curagen YTH
FBgn_Gene1_BD
FBgn_Gene2_AD

Gene1_interactions_as_BD
Gene1_interactions_total
Gene2_interactions_as_AD
Gene2_interactions_total
Screen
Reference
Release_Date
cdna
collection
hexpert
yexpert
cexpert
Curagen_confidence
ISTs_RFCs
Data_Version
Hybrigenics YTH
FBgn_Gene1_BD
FBgn_Gene2_AD

Gene1_interactions_as_BD
Gene1_interactions_total
Gene2_interactions_as_AD
Gene2_interactions_total
Screen
Reference
Release_Date
IST
ISTs_RFCs
Data_Version
PMID
URL_PubMed
